# Anteriorly Placed Midline Intraprostatic Cyst Resulting in Bothersome Lower Urinary Tract Symptoms: A Case Report

**DOI:** 10.7759/cureus.21209

**Published:** 2022-01-13

**Authors:** Fouad Hajji, Abderrazak Benazzouz, Yassine Karmouch, Nabil Hammoune, Omar Ghoundale

**Affiliations:** 1 Department of Urology, Ibn Sina Militlary Hospital/Caddi Ayyad University, Marrakesh, MAR; 2 Department of Urology, Ibn Sina Military Hospital/Caddi Ayyad University, Marrakesh, MAR; 3 Department of Radiology, Ibn Sina Military Hospital/Caddi Ayyad University, Marrakesh, MAR

**Keywords:** transurethral marsupialization, lower urinary tract symptoms, bladder outlet obstruction, bladder neck, anterior midline prostatic cyst

## Abstract

Prostatic cysts are rare, usually asymptomatic and detected incidentally at imaging. Midline prostatic cysts are less common and mostly located posteriorly. We describe a case of a 51-year-old man with unknown comorbidities who presented with troublesome irritative and voiding symptoms. Transabdominal and transrectal ultrasound, abdominal computed tomography and pelvic magnetic resonance imaging revealed a midline intraprostatic cyst protruding into the bladder lumen, with no communication with the urethra on voiding cystourethrography. Uroflowmetry findings suggested an obstructed flow. The patient underwent transurethral marsupialization of the cyst, which was found to project on the bladder outlet causing urinary obstruction, with excellent outcomes. What makes this case further interesting is the unusual cyst’s relationship with the patient’s prostate and bladder neck. To our best knowledge, this is so far the seventh reported case in the literature to describe an anteriorly placed midline intraprostatic cyst projecting at the bladder neck region and causing bothersome lower urinary tract symptoms.

## Introduction

Prostatic cysts are uncommon, observed in 0.5 to 7.9% of patients. They have mostly an embryological origin or both cause-and-effect relationship with chronic prostatitis [1, 2]. Besides their origin, they can be categorized based on their location, shape, size, interconnection with the prostatic urethra or seminal vesicles and presence of sperm in the cyst. They may be classified as isolated midline prostatic cysts (MPCs), cysts of the ejaculatory duct, simple or multiple cysts of the parenchyma, complicated cysts (infectious or hemorrhagic), cystic tumors and cysts secondary to parasitic disease [1]. Isolated MPCs are less common, and usually located posteriorly. Most cases remain unnoticed or are incidentally detected at imaging [2,3]. This report describes an unusual case of an isolated MPC, which arose from the anterior aspect of the bladder neck and presented with bothersome lower urinary tract symptoms (LUTS).

## Case presentation

A 51-year-old man presented with increased urinary urgency, frequency, hesitancy, poor intermittent stream and dysuria of 15 months’ duration. He denied any history of pelvic or perineal trauma, urethral instrumentation, urological disease or neurological deficit. He described his LUTS as moderate and stated that he has been extremely bothered over the last few months. He had three children and did not have any complaint of gross hematuria, urethral discharge, erectile dysfunction, haematospermia or post-coital discomfort, other ejaculatory disorders or infertility.

Digital rectal examination revealed a smooth, normally firm, and non-tender prostate gland. Urinalysis did not show microscopic hematuria, culture was sterile, and cytology was negative for malignancy. The serum-specific prostatic antigen level was 0.51 ng/mL and routine blood tests were within normal limits. Uroflowmetry revealed a maximal flow rate of 10 mL/s with a flat curve, voided volume of 290 mL, voiding time of more than 2 minutes, and the postvoid residual (PVR) urine volume was 90 ml, suggesting an obstructed flow.

**Figure 1:**
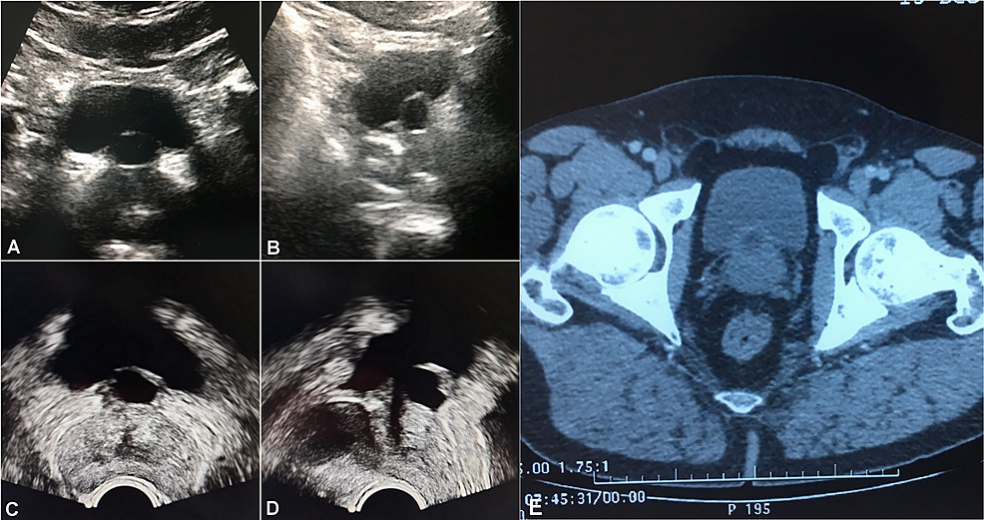
Ultrasound-CT image showing an anteriorly placed midline prostatic cyst projecting at the bladder neck region. (A) Axial and (B) Sagittal transabdominal ultrasound images. (C) Axial and (D) Sagittal TRUS images. (E) Pelvic axial CT image. TRUS:  transrectal ultrasonography

**Figure 2:**
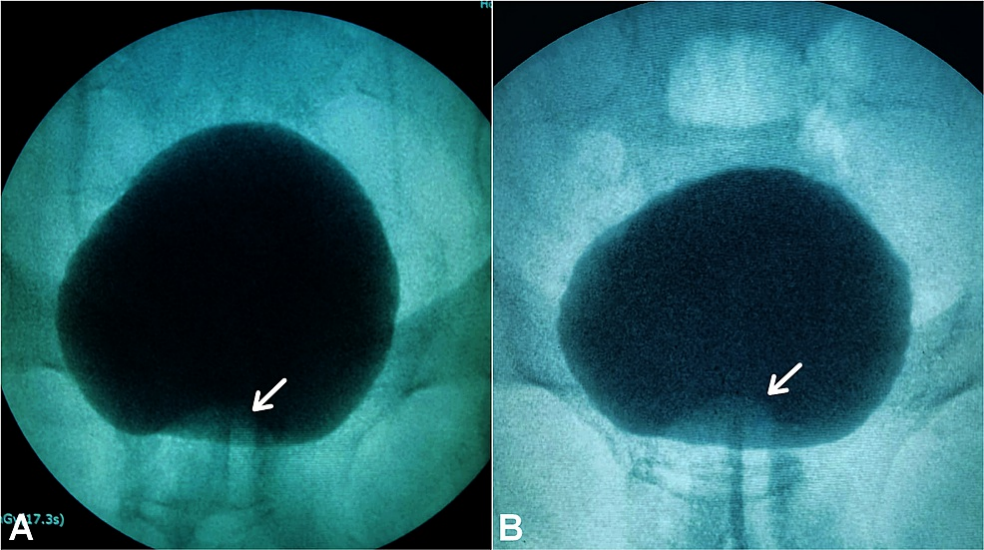
Intravesical protrusion of a midline prostatic cyst (white arrows). (A) Excretory urogram image showing no evidence of ectopic ureterocele. (B) VCUG image showing no evidence of communication with the prostatic urethra. VCUG: voiding cystourethrography

Abdominal and transrectal ultrasonography (TRUS), and abdominal computed tomography (CT) showed an isolated, anteriorly positioned and midline intraprostatic cystic lesion with an approximate diameter of 1.5 cm that projected at the level of the bladder neck region (Figure 1). On TRUS, the volume of the prostate gland was approximately 30 mL (4.4 x 3.6 x 3.8 cm). Excretory urography and voiding cystourethrography (VCUG) done subsequently showed no evidence of ectopic ureterocele and did not demonstrate any communication with the prostatic cyst, respectively (Figure 2).

**Figure 3:**
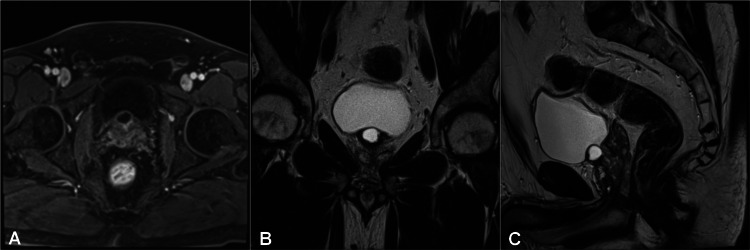
Pelvic MRI image showing an anteriorly positioned midline prostatic cyst projecting at the bladder neck region with no evidence of communication with the urethra. (A) Axial T1-weigthed MRI image. (B) Coronal and (C) Sagittal T2-weighted MRI images.

In order to investigate further, pelvis magnetic resonance imaging (MRI) revealed an isolated anteriorly placed MPC of 1.7 x 1.5 x 1.3 cm arising from the upper part of the bladder neck region and protruding into the bladder lumen, with no evidence of any communication with the urethra (Figure 3). There was no evidence of extraprostatic extension, pelvic lymphadenopathy or other associated genitourinary abnormalities.

The patient was told his complaints were more likely to be related to the cyst. However, a benign prostatic obstruction could not be ruled out despite his small-sized prostate. The patient was started on alpha blocker drug treatment, with a plan to undergo transurethral resection of the cyst if the medical therapy proved to be ineffective. After four weeks, his symptoms had not improved and he consented to undergo transurethral marsupialization of the cyst.

**Figure 4:**
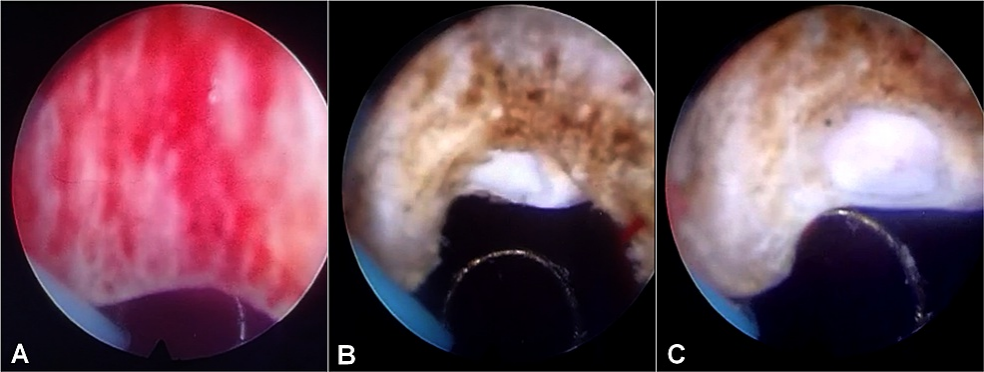
Intraoperative view showing transurethral marsupialization of an anteriorly located MPC. (A) Backward bulging of the anterior aspect of the bladder neck. (B) Limited resection of the prostatic tissue at precisely twelve o’clock with incision of the cyst at its base. (C) Incomplete marsupialization of the cyst into the bladder neck lumen. MPC: midline prostatic cyst

Under general anesthesia, an initial cystoscopic examination showed an anterior bulge of the bladder neck region protruding backwards to obstruct the bladder outlet (Figure 4A), with no evidence of medial or lateral lobe enlargement. Due to the difficult position of the cyst and the potential risk of retrograde ejaculation, a limited resection of the prostatic tissue at precisely twelve o’clock was required (Figure 4B). Then, the cyst was incised only at its base and incompletely marsupialized into the bladder neck lumen with a wire loop to discharge a transparent fluid from its cavity (Figure 4C).

**Figure 5:**
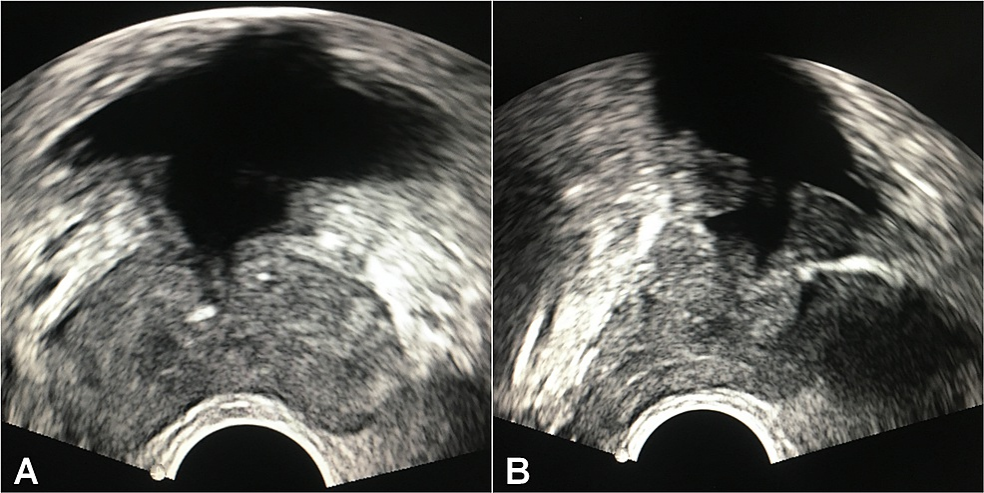
TRUS image showing a residual cystic cavity at the bladder neck region. (A) Axial TRUS image. (B) Sagittal TRUS image. TRUS:  transrectal ultrasonography

The postoperative period was uneventful. The catheter was then removed and the patient was discharged the next morning. Fortunately, the patient’s symptoms improved dramatically. Histopathology of the cystic wall did not show any evidence of malignancy. A TRUS, done four weeks after, showed a large residual cystic cavity at the bladder neck region (Figure 5). At his twelve-month follow-up, the patient denied any voiding difficulties, ejaculatory disorders or other sexual dysfunctions, and had neither obstructed flow on uroflowmetry nor recurrence on TRUS.

## Discussion

MPCs are rare, with an incidence of less than 1% [3]. Owing to the widespread availability and routine use of TRUS, CT and MRI, there is currently a continued pattern of increase in reported cases with an estimated prevalence of 5-14 % [4]. Most cases are posteriorly located and asymptomatic. They may occasionally get infected, serve as a site for recurrent infections, be significantly enlarged to compress the nearby structures; the bladder neck, the urethra or the ejaculatory duct [3, 4]. Hence, MPCs may be associated with recurrent urinary tract infections, prostatitis, epididymitis, chronic pelvic pain syndrome, hematuria, haematospermia, low semen volume and even infertility. Moreover, MPCs are reported to occur in 5% of patients with LUTS, to cause urinary retention or to be confused with benign prostatic hyperplasia (BPH) or neuropathic bladder [1, 3-5].

Anteriorly placed MPCs presenting with LUTS are extremely rare since only about 10 reports have ever been published [3,4, 6-12]. Some cases have been reported to arise at the level of prostatic urethra, causing bladder outlet obstruction and being managed conservatively with antegrade ejaculation preservation [9-12]. What makes the present case further interesting is the unusual location of the MPC at the anterior aspect of the bladder neck region, raising both diagnostic and therapeutic challenges. To our best knowledge, this is so far the seventh case of its kind to be reported in the literature.

MPCs are mostly developmental in origin and represent a common variant in 7.6% of healthy men [1, 2]. They are traditionally be classified as Müllerian duct and prostatic utricle cysts, and are of mesodermal or endodermal origin respectively. Unlike Müllerian duct cysts, utricular cysts communicate with the urethra, do not extend above the base of the prostate, may contain sperm, are usually diagnosed in childhood (peak of incidence < 20 years of age) and are frequently associated with various genitourinary abnormalities [2, 13]. The present case, with an isolated non-communicating MPC arising at the base of the prostate to form a visible protrusion into the bladder lumen in an adult, may be considered as a müllerian duct cyst. However, not all MPCs are Müllerian duct cysts or prostatic utricle cysts [13], and the possibility of other cystic lesions should be considered (i.e. prostatic retention cysts). Nevertheless, the etiology of MPCs may have no clinical relevance as they all have similar presentations and treatments.

MPCs may be easily detected on transabdominal or TRUS, CT and MRI [3, 4]. In this case, excretory urogram and VCUG were helpful in primarily ruling out ectopic ureterocele and enlarged prostatic utricle cyst, respectively. However, an individual diagnosed with such a condition should undergo a focused diagnostic workup to determine whether the cyst represents a normal variant or is etiologically involved in the patient’s complaints.

In this case, with an irrelevant past medical history, there were no clinical symptoms, physical signs or radiological/endoscopic evidence of prostatic hyperplasia, primary bladder neck obstruction, urethral stricture or neurologic bladder. Whereas, the cystoscopic examination had revealed an anterior swelling of the bladder neck bulging posteriorly to obstruct the bladder outlet, supporting uroflow findings. Hence, the cyst was thought to act like a prostatic median lobe with a consequent intravesical protrusion, distorting the laminar flow at the bladder neck in a checking valve manner and irritating the bladder respectively [7]. Indeed, the presence of MPCs alone does not predict the development of moderate to severe LUTS, unless there are anatomically projecting to the bladder outlet and subsequently causing outlet obstruction [14], as in the current patient. However, cases with noncontributory endoscopic findings to demonstrate this physical ball-valve phenomenon may undergo transrectal aspiration of the cyst as a safer approach before surgical management to establish the cause-and-effect relationship with the patient’s symptoms.

There are currently no guidelines on the management of prostatic cysts. Asymptomatic patients should be followed without intervention. When symptomatic, active intervention is recommended and changes according to the cyst’s location and size, and associated conditions. It can include transrectal aspiration with or without sclerotherapy, transurethral resection of the prostate (TRUP), transurethral and percutaneous unroofing or marsupialization of the cyst using wire loop, Collin’s knife or Holmium laser, and less frequently, open surgery [3-9, 15]. The present case could have been managed by transrectal aspiration of the cyst, but potential risk factors include infection and recurrence. Indeed, durable recurrence-free results have been reported for MPCs treated with transurethral approach [3-7, 9]. In this case, unroofing the cyst can preserve the bladder neck and the antegrade ejaculation, but may be technically challenging via a transurethral approach when located anteriorly. Consequently, transurethral marsupialization may be a feasible and effective treatment option for small anteriorly located MPCs (2 cm x 2 cm) [4,7], as in this case. However, it is important to distinguish MPCs originating in the bladder neck region from infravesical cysts, as the latter may be marsupialized into the urethra with a lower risk of causing retrograde ejaculation [9-12].

Our intraoperative observation of incomplete marsupialization of the cyst, which was located in a challenging position to be safely unroofed via a transurethral route, supports Al-Nasser and Almannie's recommendation [3, 15]. Accordingly, a percutaneous approach could be superior to a transurethral marsupialization, providing an efficient, safe, and successful procedure to unroofing an anteriorly placed MPCs projecting at the bladder neck region. Furthermore, its growing utilization to treat large bladder stones and BPH may certainly lead to increasingly routine use in the near future in such situations. Hence, we will strongly recommend this approach to our patient in case of symptomatic recurrence of his cyst. Regardless of the approach chosen, pathological examination of the cyst’s wall is important, since malignant transformation was reported in association with such MPCs, although anecdotal [13].

## Conclusions

MPC is an uncommon condition and mostly asymptomatic, yet it should be considered as a differential diagnosis in men with LUTS. Anteriorly placed MPCs are extremely rare, but their surgical management may be technically challenging and harbor the potential risk of retrograde ejaculation as most cases arise from the bladder neck region. Hence, an individual diagnosed with such a condition should undergo a focused diagnostic workup to establish the cause-and-effect relationship with the patient’s complaints. When symptomatic, the cyst should be managed conservatively. Transurethral marsupialization of the cyst can be used in selected cases (i.e. infravesical MPC), whereas, the percutaneous approach could be superior to transurethral techniques, providing efficient, more secure, and effective treatment option to unroofing an anteriorly placed MPCs protruding into the bladder lumen, especially when approaching symptomatic and sexually active young patients wishing to preserve ejaculation and fertility.
